# Type I/II Interferon in HIV-1-Infected Patients: Expression in Gut Mucosa and in Peripheral Blood Mononuclear Cells and Its Modification upon Probiotic Supplementation

**DOI:** 10.1155/2018/1738676

**Published:** 2018-08-12

**Authors:** Claudia Pinacchio, Giuseppe Corano Scheri, Maura Statzu, Letizia Santinelli, Giancarlo Ceccarelli, Giuseppe Pietro Innocenti, Vincenzo Vullo, Guido Antonelli, Jason M. Brenchley, Gabriella d'Ettorre, Carolina Scagnolari

**Affiliations:** ^1^Department of Public Health and Infectious Diseases, Sapienza University of Rome, Viale del Policlinico 155, 00161 Rome, Italy; ^2^Department of Molecular Medicine, Laboratory of Virology, Sapienza University of Rome, Viale di Porta Tiburtina 28, 00185 Rome, Italy; ^3^Istituto Pasteur Italia, Fondazione Cenci Bolognetti, Viale Regina Elena 291, 00161 Rome, Italy; ^4^Laboratory of Viral Diseases, National Institute of Allergy and Infectious Diseases, NIH, Bethesda, MD 20892, USA

## Abstract

Expression of type I and II interferon (IFN) was evaluated in gut-associated lymphoid tissue (GALT) and peripheral blood mononuclear cells (PBMCs) of HIV-1-positive patients on long-term, suppressive, antiretroviral therapy before and after probiotic supplementation. IFN*α* subtypes and IFN*β* were expressed at higher levels in GALT compared to PBMC, whereas an opposite trend of expression was recorded for IFN*γ*. An increase of IFN*α*6, IFN*α*10, IFN*α*14, IFN*α*17, and IFN*α*21 and a decrease of IFN*γ* were observed in both anatomical sites after probiotic supplementation.

## 1. Introduction

A strong relationship between type I interferon (IFN) response and disease progression in chronic HIV-1 and simian immunodeficiency virus (SIV) infection exists [1]. IFN*α* was recently identified as the predominant type I IFN expressed in plasma during untreated, chronic HIV-1 infection [2]. However, IFN*α* is not a unique cytokine; it consists of a group of at least 12 structurally related subtypes with specific biological activity and which might be differentially expressed during viral infection [3]. A recent comprehensive direct study of IFN*α* subtype expression in HIV-1 infection and of the type I IFN signature in specific anatomical sites, such as the gastrointestinal tract, showed a compartmentalized IFN-I response during chronic untreated HIV-1 infection, with IFN*β* being more predominant in the gut. [4]. In this regard, it is not completely clear whether constitutive type I IFN production in the intestine is driven by commensal microbial signals and/or modulated by dietary, probiotic, and prebiotic interventions. However, the protective effects of commensal and probiotic bacteria in the intestine have been shown to be mediated, in part, by the induction of type I IFN, and, more importantly, IFNAR1−/− mice have increased susceptibility to dextran sodium sulfate-induced acute colitis [5, 6].

Thus, this topic deserves investigation as a gastrointestinal dysfunction associated with altered microbiome composition and a severe enteropathy is one of the hallmarks of HIV-1 pathogenesis [7].

Given that type I IFN is thought to have detrimental effects during HIV-1 infection and that each IFN*α* subtype displays different anti-HIV-1 activity [8], the expression of all IFN*α* subtypes, IFN*β*, and IFN*γ* was evaluated both in gut-associated lymphoid tissue (GALT) and peripheral blood mononuclear cells (PBMCs) of antiretroviral therapy- (ART-) treated HIV-1 patients. Moreover, since probiotics have shown promising effects in improving gut function in HIV-1 subjects [7], their role in modulating GALT- and PBMC-associated type I and II IFN expression was analyzed in ART-treated HIV-1 patients after several months of a twice daily dietary supplement with a multistrain probiotic formulation [9].

## 2. Material and Methods

### 2.1. Patients

Ten Caucasian HIV-1-positive patients on long-term suppressive ART were recruited at the Division of Infectious Diseases, Department of Public Health and Infectious Diseases, Hospital of “Sapienza” University of Rome (Italy). All HIV-1-infected patients received a high concentration of lyophilized multistrain probiotic supplement (*Lactobacillus plantarum* DSM 24730, *Streptococcus thermophilus* DSM 24731, *Bifidobacterium breve* DSM 24732, *Lactobacillus paracasei* DSM 24733, *Lactobacillus delbrueckii* subsp. *bulgaricus* DSM 24734, *Lactobacillus acidophilus* DSM 24735, *Bifidobacterium longum* DSM 24736, and *Bifidobacterium infantis* DSM 24737) twice a day for six months [9, 10]. This formulation is commercialized as Vivomixx in EU, Visbiome in USA, and the DeSimone Formulation in Korea [9]. The probiotic preparation was administered per os at a daily dosage of 1.8 × 10^12^ live bacteria. The study was approved by the institutional review board (Sapienza University of Rome), and all study participants gave written informed consent.

### 2.2. Laboratory Procedures and Analysis Sampling

Patients were sampled for peripheral blood (20 mL) and underwent endoscopic procedures. Colonic washing was carried out by PEG administration 24 hours before the examination. The endoscopic procedure was performed with conscious sedation (midazolam 5 mg/iv) using large cup forceps (Radial Jaw 4, Boston Scientific, Natick, MA, USA). All HIV-1-positive patients underwent a total colonoscopy and retrograde ileoscopy for at least 10 cm of distal ileum with conventional or slim colonoscope (model CF or PCF 160 AI, Olympus Medical Europe GmbH, Hamburg, Germany). We obtained specimens from the terminal ileum, cecum, ascending, transverse, and descending colon. Peripheral blood mononuclear cells (PBMCs) and lamina propria lymphocytes (LPLs) were stored as dried pellets for RNA extraction and subsequent evaluation of IFN*α* subtypes, IFN*β*, and IFN*γ* levels.

### 2.3. PBMC and LPL Processing

Peripheral blood samples were collected in tubes containing ethylene-diamine-tetraacetic acid (EDTA), and plasma was previously separated by centrifugation. Blood was processed to obtain PBMCs by Ficoll gradient centrifugation (Lympholyte, Cedarlane Labs, Hornby, Ontario, Canada). Gut biopsies from each intestine site were pooled and processed. Briefly, biopsies collected in RPMI 1640 were washed twice with EDTA wash media, resuspended, and incubated for 1 hour at room temperature in 5 mM EDTA solution. Supernatant containing intraepithelial lymphocytes was removed, and biopsies were digested by 1-hour incubation at 37°C with 1 mg/mL collagenase (Sigma-Aldrich, Milan, Italy) and 1.5 U DNAse I (Sigma-Aldrich, Milan, Italy), allowing the isolation of LPLs that were filtered through a 70 *μ*m cell strainer.

### 2.4. TaqMan-Based Real-Time RT-PCR Assays for mRNA Expression

Quantitative real-time PCR for IFN*α* (*n* = 12), IFN*β*, and IFN*γ* was carried out with the LightCycler 480 instrument (Roche, Basel, Switzerland). Briefly, total RNA was extracted from PBMCs and LPLs using the RNeasy Plus Universal Tissue Mini Kit (Invitrogen, Carlsbad, CA, USA) and reverse transcribed using the High Capacity cDNA Reverse Transcription Kit (Applied Biosystems, USA), according to the manufacturer's protocol. Primers and probes for each gene were added to the Probes Master Mix (Roche, Basel, Switzerland) at 500 and 250 nM, respectively, in a final volume of 20 *μ*L. The housekeeping gene *β*-glucuronidase [11] was used as an internal control. Gene expression values were calculated by the comparative Ct method. The primers and probe were assayed on demand and were purchased from Integrated DNA Technologies (IDT), Iowa, USA. The list of primers and probes is as follows: IFN*α*1 (Hs.PT.58.46311748.g), IFN*β* (Hs. PT.58.39481063.g), IFN*α*2 (Hs.PT.58.24294810.g), IFN*α*4 (Reference number: 68098028), IFN*α*5 (Hs.PT.58.39565646.g), IFN*α*6 (Hs.PT.58.40193986.g), IFN*α*7 (Hs.PT.58.25568785.g), IFN*α*8 (Hs.PT.58.40433689.g), IFN*α*10 (Hs.PT.58.24640720.g), IFN*α*14 (Reference number: 68098032), IFN*α*16 (Hs.PT.58.1479042.g), IFN*α*17 (Reference number: 68098036), IFN*α*21 (Hs.PT.58.45746476.g), and IFN*γ* (Hs.PT.58.3781960.g).

### 2.5. Statistical Analysis

Data are expressed as median/range. Differences in the levels of type I and II IFN genes between GALT and PBMC were evaluated using the Wilcoxon test. The same test was used to evaluate changes in all type I and II IFN genes before and after six months of probiotic supplementation. Differences were considered statistically significant when *p* < 0.05. All analyses were performed with the SPSS v.17.0 for Windows.

## 3. Results

Transcript levels of several IFN*α* subtypes (*n* = 12), IFN*β*, and IFN*γ* both in PBMC and GALT were measured in 10 ART-treated HIV-1-infected patients (gender: 100% males, age (median/range): 42/22–53 years, CD4+ T cell count (median/range): 674/564–824 cells/mm^3^, HIV-1 RNA: <37copies/mL, duration of antiretroviral therapy (median/range): 6/1.75–16.25 years).

**Figure 1:**
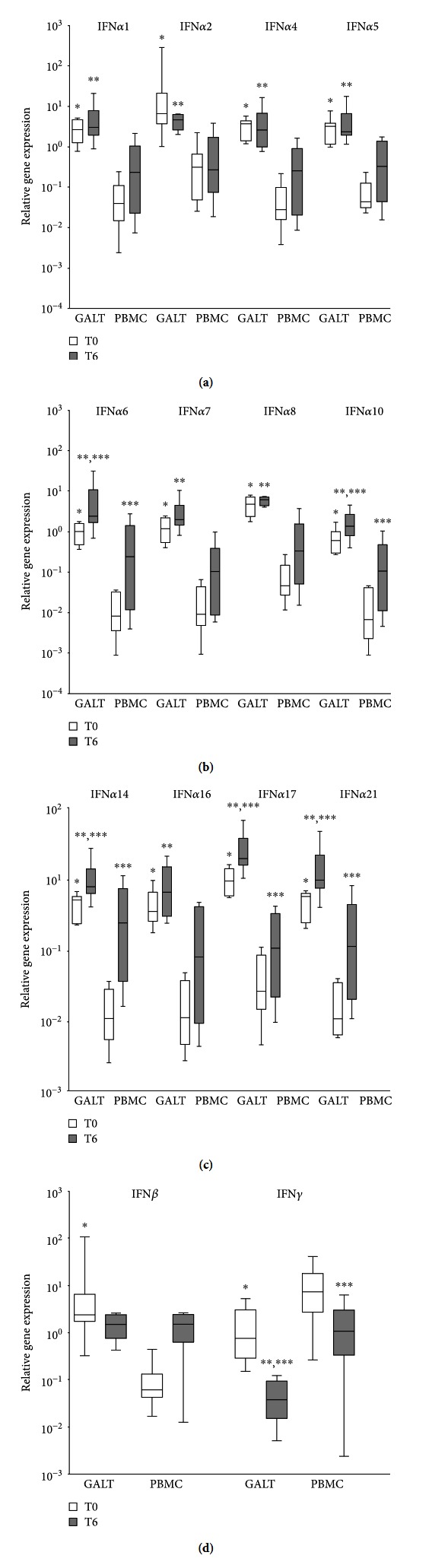
Expression of IFN*α* subtypes (*n* = 12), IFN*β*, and IFN*γ* in gut-associated lymphoid tissue (GALT) and peripheral blood mononuclear cells (PBMCs) from treated HIV-1-positive patients before (T0, white colour) and after 6 months (T6, grey colour) of probiotics supplementation (*n* = 10). Panels (a–d) data were analyzed using the Wilcoxon test. Differences were considered statistically significant when *p* < 0.05. ^∗^ Significant differences (*p* < 0.05) between GALT and peripheral blood (T0); ^∗∗^ significant differences (*p* < 0.05) between GALT and peripheral blood (T6); ^∗∗∗^ significant differences (*p* < 0.05) between T0 and T6.

**Figure 2:**
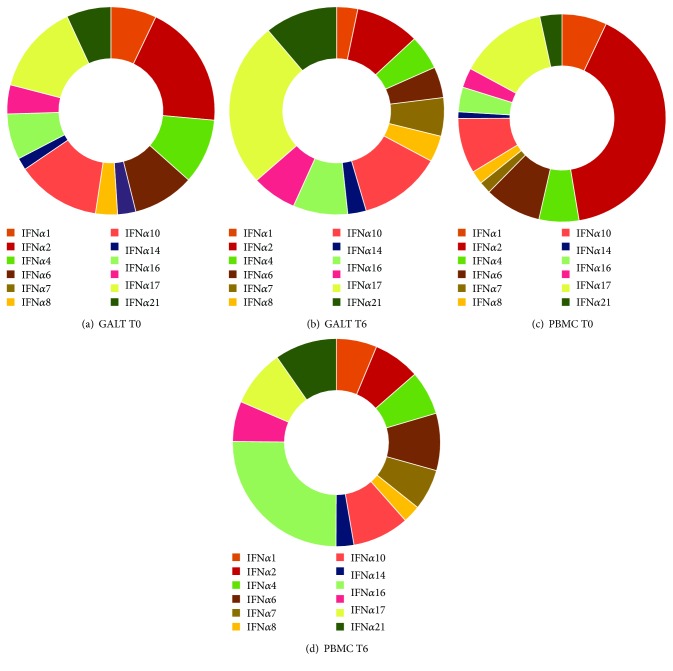
IFN*α* subtype signature in gut-associated lymphoid tissue (GALT) and peripheral blood mononuclear cells (PBMCs) from treated HIV-1-positive patients before (T0) and after 6 months (T6) of probiotic supplementation. Pie chart represents the expression of each IFN*α* subtype. Data are expressed as percentage.

Levels of all IFN*α* subtypes and IFN*β* were higher in GALT than in PBMC (*p* < 0.05 for all genes, Figures 1(a)–1(d)). Specifically, an average of 50-fold increase of IFN*α*/*β* subtypes in GALT compared to PBMC was recorded. The following type I IFN exhibited the highest and lowest differences between the two anatomical sites analyzed: IFN*α*21 (72 times) and IFN*α*2 (17 times). Conversely, IFN*γ* gene expression was higher (approximately 14-fold) in PBMC compared with GALT (Figure 1(d)). Although a different IFN*α* subtype expression pattern was observed in GALT and peripheral blood, some similarities in type I IFN signature have emerged (Figure 2). In particular, the highest and lowest IFN*α* subtypes expressed were IFN*α*2 and IFN*α*6/*α*7, *α*10, respectively.

We found that IFN*α* subtypes, IFN*β*, and IFN*γ* transcript levels as well as the IFN*α* subtype expression profile changed after probiotic supplementation (Figures 1(a)–1(d)). In particular, IFN*α*6, IFN*α*10, IFN*α*14, IFN*α*17, and IFN*α*21 significantly increased after probiotic treatment in both GALT and PBMC of HIV-1-positive patients (Figures 1(b) and 1(c)). Interestingly, by contrast to what observed for IFN*α*6, IFN*α*10, IFN*α*14, IFN*α*17, and IFN*α*21 subtypes, IFN*γ* levels decreased significantly in both anatomical sites after probiotic supplementation (Figure 1(d)). For the other type I IFN genes analyzed, we observed a reduction or an increment depending on the specific type I IFN considered, but the differences did not reach statistical significance.

## 4. Discussion

Four major points emerged from our current analysis of type I/II IFN response in GALT and PBMC of HIV-1-infected patients before and after probiotic treatment: (1) all IFN*α* subtypes and IFN*β* are more strongly expressed in GALT than in PBMC whereas IFN*γ* exhibits an opposite trend; (2) IFN*α* subtype expression signature in GALT is different from the signature in PBMC; (3) levels of the IFN*α* subtypes and their signature significantly change after probiotic supplementation; and (4) probiotic supplementation is associated with a decrease of the IFN*γ* levels. Overall, the results are very clear; their interpretation however requires much attention and caution.

The very high endogenous expression of all IFN*α*/*β* subtypes in GALT could be sustained by the chronic stimulation of pattern recognition receptors by intestinal bacteria ligands which subsequently drive the production of type I IFN [6]. The levels of IFN*α* subtypes and IFN*β* could also be enhanced in a situation of gut microbiota dysbiosis in HIV-1-positive patients, where there is an expansion of the species belonging to the Proteobacteria phylum [12–14]. Even specific, and/or previously undescribed viruses, within the gut virome, might contribute to the production of type I IFN [15].

Regardless of the differences in the relative abundance of each IFN*α* subtype between the two anatomical sites, it is also important to emphasize that the profile of IFN*α* subtype expression differs in GALT and PBMC during HIV-1 infection. A distinct profile of IFN*α* subtype expression could suggest that not all subtypes play a negative role in chronic HIV-1 infection. Directly related to the latter, we found that probiotic supplementation can modulate type I IFN subtype expression differentially, causing a significant induction of IFN*α*6, IFN*α*10, IFN*α*14, IFN*α*17, and IFN*α*21 in both the GALT and PBMC of HIV-1-infected patients. Interestingly, four of these IFN*α* subtypes, IFN*α*6, IFN*α*14, IFN*α*17, and IFN*α*21, exhibited a more potent antiviral activity against HIV-1 compared to other subtypes [8]. In particular, IFN*α*14 had the ability to reduce both viremia and proviral loads *in vivo* [8, 16], while another study on mice has shown the potency of IFN*α*8, IFN*α*14, and IFN*α*6 in inhibiting productive HIV-1 infection [17].

It is also noteworthy that a preferential upregulation of IFN*α*2 was recorded in both anatomical sites. In agreement, the IFN*α*2 subtype was the most strongly expressed in the PBMC and plasmacytoid dendritic cells of HIV-1 patients [18], although this subtype exhibited relatively weak anti-HIV-1 activity [8].

Conversely, a reduction of IFN*γ*, which has an opposite trend of expression to IFN*α*/*β* subtypes in GALT and PBMC, was observed after probiotic treatment in both the anatomical sites analyzed. A significant steadily increasing trend in IFN*γ* levels in chronic progressive HIV-1 disease has been demonstrated [19], suggesting that probiotics can also improve this immunologic response in HIV-1-infected patients. The lack of a comparison of the IFN levels between HIV-1-infected patients and a healthy control represents a limitation of this pilot study. This analysis is needed to ascertain whether changes in IFN expression might be beneficial or detrimental to HIV-1-positive patients. However, the reduction of IFN*γ* levels in both PBMC and GALT after supplementation could serve as a suggestive control indicator that the probiotic is behaving beneficially for HIV-1-infected patients. Indeed, an excess of mucosal mRNA expression of IFN*γ* has been reported to be associated with high levels of HIV-1 replication and profound CD4+ T-cell depletion [20].

This finding was also strongly supported by our previous studies on the same probiotic formulation, in which its ability to reduce several biomarkers of inflammation was showed. In particular, the probiotic product employed in our study, when supplemented for 6 months in HIV-1-positive patients, was associated with a significant reduction of GALT-associated indoleamine 2,3-dioxygenase (IDO) mRNA levels. This enzyme plays a key role in the tryptophan metabolism and is involved in the chronic immune activation status of patients with HIV-1 infection [9, 21]. Moreover, a reduction in the frequencies of CD4+ and CD8+ T-cell subsets, expressing CD38+, HLA-DR+, or both, and an increase in the percentage of Th17 cell subsets, especially those with central or effector memory phenotype, were recorded in the peripheral blood and in GALT after the same probiotic intervention [10]. Probiotic supplementation was also associated to a recovery of the integrity of the gut epithelial barrier, a reduction of both intraepithelial lymphocytes density and enterocyte apoptosis and an improvement of mitochondrial morphology sustained in part by a modulation of heat shock protein 60 [10].

Altogether, the results of this study should be taken with caution since it is a pilot, nonrandomized single arm, clinical study on the effects of this specific probiotic on type I and II IFN response in HIV-1-infected patients. Nevertheless, it provides the first evidence that all IFN*α* subtypes, IFN*β*, and IFN*γ* are differentially expressed in the GALT and PBMC of ART-treated HIV-1-infected patients, and this multistrain probiotic supplementation can change the expression of some IFN*α* subtypes and IFN*γ*, highlighting the important role of gut microbiome composition in regulating the type I and II IFN response and providing the basis for a well-executed large clinical trial, including healthy controls. Moreover, since HIV-1 infection is characterized by a persistent immune activation [22], the analysis of IFN response concomitant with measures of well-established immunological markers (e.g., interleukin-6 (IL-6), lipopolysaccharide binding protein (LBP), and CD4 counts) should be performed in HIV-1-infected patients to ascertain the potential benefit of this probiotic supplementation.

## Data Availability

In order to comply with Italian law and regulations on patients' privacy, data are not publicly available but can eventually be made available upon request.

## Abbreviations

IFN: Interferon
GALT: Gut-associated lymphoid tissue
PBMCs: Peripheral blood mononuclear cells
SIV: Simian immunodeficiency virus
ART: Antiretroviral therapy
LPL: Lamina propria leukocytes
EDTA: Ethylene-diamine-tetraacetic acid.
